# Evidence for Genetic Variation in Human Mate Preferences for Sexually Dimorphic Physical Traits

**DOI:** 10.1371/journal.pone.0049294

**Published:** 2012-11-14

**Authors:** Karin J. H. Verweij, Andrea V. Burri, Brendan P. Zietsch

**Affiliations:** 1 School of Psychology, University of Queensland, Brisbane, Queensland, Australia; 2 Genetic Epidemiology Laboratory, Queensland Institute of Medical Research, Brisbane, Queensland, Australia; 3 Department of Twin Research and Genetic Epidemiology, St. Thomas’ Hospital, King’s College London, London, United Kingdom; University of Lausanne, Switzerland

## Abstract

Intersexual selection has been proposed as an important force in shaping a number of morphological traits that differ between human populations and/or between the sexes. Important to these accounts is the source of mate preferences for such traits, but this has not been investigated. In a large sample of twins, we assess forced-choice, dichotomous mate preferences for height, skin colour, hair colour and length, chest hair, facial hair, and breast size. Across the traits, identical twins reported more similar preferences than nonidentical twins, suggesting genetic effects. However, the relative magnitude of estimated genetic and environmental effects differed greatly and significantly between different trait preferences, with heritability estimates ranging from zero to 57%.

## Introduction

Expanding on ideas first proposed by Darwin [1], a number of researchers [2], [3], [4], [5], [6] have recently suggested that certain human morphological traits may have evolved via sexual selection, whereby the mate preferences of one sex affect the reproductive success of the opposite sex (i.e. intersexual selection). Such traits often differ markedly between the sexes, because the preferences of one sex can unidirectionally influence characteristics in the opposite sex. Sexually selected traits may also differ between human populations, as slight initial differences in preferences between populations can lead to rapid divergence in expression of the preferred traits [7]. Examples of sexually dimorphic morphological traits that have been hypothesised to have been affected by sexual selection include (but are not limited to) hair and skin colour, breast size, facial hair, chest hair, head hair length, and body size – these traits have been previously shown to contribute to judgements of physical attractiveness [8], [9], [10], [11], [12], [13].

The source of the preferences for these traits is important to sexual selection explanations. Darwin noted that it is “possible that certain tastes may in the course of time become inherited, though there is no evidence in favour of this belief”, and that if true it would allow for sexual selection to favour varying features in populations that have inherited different “innate ideal standard[s] of beauty” [1]. For preferences of one sex to affect the evolution of a trait in the other (i.e. all models of intersexual selection), the preferences must be exercised over evolutionary timescales, implying a genetic basis to mate preferences.

However, the source of mate preferences for human morphological traits has not been established. Sexual imprinting (i.e. the opposite-sex parent is used as a template for an ideal mate) appears important in a number of species [14], [15], but its role in humans remain uncertain [16], [17]. Animal studies suggest that genetic factors play a role in variation in mate preferences [18], but attempts to quantify such genetic variation (e.g. heritability, i.e. the proportion of total variation that is due to genetic variation) have yielded mixed results [19], [20], [21], [22], [23], [24], [25], [26], (see [27] for a review). In humans there has been much less investigation into possible genetic effects on mate preferences. Several studies suggest that individuals tend to prefer the odour of those with dissimilar human leukocyte antigen (HLA) (see [28] for a review); however, the two studies to investigate how HLA might relate to morphological preferences (for facial similarity) found significant effects in completely opposite directions [29], [30], so it remains to be established whether a true effect exists for morphological preferences. A recent twin study on unconstrained human mate preferences suggested that the importance placed on physical attractiveness (relative to other, non-physical cues of mate quality) is moderately heritable [31], but there has been no quantitative assessment of genetic influences on unconstrained human mate preferences for specific morphological traits. Human behavioural traits tend to be heritable [32], but the high specificity of preferences for highly malleable traits like head and facial hair, and their apparent fluctuation with cultural trends in beauty and fashion, makes it unclear if such preferences would have a genetic basis. Furthermore, a large study of twins and their romantic partners suggests very low (nonsignificant) genetic variation in realised mate choice (i.e. actual partnerships) for height and body mass index (along with other, non-morphological traits) [17]. However, we cannot necessarily extrapolate these latter results for realised mate choice to unconstrained mate preferences because the relationship between preferences and actual partnership formation is poorly understood [33].

Here we use a large twin sample to investigate genetic and environmental influences on forced-choice, dichotomous preferences for height, skin colour, hair colour and length, chest hair, facial hair, and breast size. It should be noted that while demonstrating significant heritability would indicate a genetic basis to these preferences, lack of significant heritability would not necessarily indicate the lack of a genetic basis, since a genetic basis could be invariant in the population, which would not contribute to heritability [34].

## Methods

### Ethics Statement

The study was approved by the St. Thomas’ Hospital research ethics committee. All study participants involved in this study provided informed written consent.

### Participants

Data were from 4,586 twin individuals aged 19 to 83 from the UK Adult Twin Registry, a cohort of unselected volunteer Caucasian twins. The sample consists mostly of females because initial research focused on diseases with higher prevalence in women than in men. There were 4,044 females (mean age = 51.0±12.7) and 541 males (mean age = 49.2±14.0), including 1,762 full pairs (49.1% identical; monozygotic; MZ, and 50.9% nonidentical; dizygotic; DZ) and 1,060 single twins. There were too few (16) opposite sex pairs for stable correlation estimates, so these individuals were treated as single twins. Further details on the data collection and zygosity determination, and on the comparability of the twins to age-matched singleton populations can be found elsewhere [31], [35], [36]. Full ethical approval has been granted and participants’ consent has been obtained. .

### Measures

Participants reported their preferred features in a partner with dichotomous response options (see Table 1). Between 6 and 12% of data points on each trait preference were missing.

**Table 1:** Frequencies for dichotomous mate preferences for morphological traits.

		Proportion preferring each trait	
		Women	Men
**Height**	Tall	0.90	0.53
	Short^a^	0.10	0.47
**Skin colour**	Fair skin^a,b^	0.90	0.39
	Olive skin	0.10	0.61
**Hair colour**	Blond hair^a^	0.21	0.39
	Brown hair	0.79	0.61
**Hair length**	Short^a,b^	0.55	0.63
	Long	0.45	0.37
**Chest hair**	Hairy chest	0.40	–
	Smooth chest^a^	0.60	–
**Facial hair**	Beard/moustache^a^	0.13	–
	Clean-shaven	0.87	–
**Breast size**	Large breasts	–	0.57
	Small breasts	–	0.43

Superscript ‘a’ (‘b’) indicates older women (men) were significantly (i.e. p<.05) more likely to prefer this trait than younger women (men).

### Analyses

In accordance with standard genetic analysis of twin data, all analyses employed maximum-likelihood modelling procedures using the statistical package Mx [37] and assumed that a threshold delimiting the dichotomous preference categories overlayed a normally distributed continuum of liability. In maximum-likelihood modelling, the goodness-of-fit of a model to the observed data is distributed as chi-square (χ^2^). By testing the change in chi-square (Δχ^2^) against the change in degrees of freedom (Δdf), we can test whether dropping or equating specific model parameters significantly worsens the model fit, and can thus test hypotheses regarding those parameters.

Variance in the preferences was partitioned into that due to additive genetic (A), nonadditive genetic (D), shared (family) environmental (C), and residual influences (E). This can be achieved because MZ twins share all of their genes, while DZ twins share on average only half of their segregating genes. A, D, C, and E influences predict different patterns of MZ and DZ twin correlations, and structural equation modelling is used to determine the combination of influences that best matches the observed data. A limitation of the classical twin model is that there is not enough information to estimate C and D simultaneously; C is estimated if the DZ twin correlation is more than half the MZ twin correlation, and D is estimated if the DZ twin correlation is less than half the MZ correlation. Further details of the twin design, including assumptions, can be found elsewhere [38], [39]. Analyses were performed separately for each sex. Age was controlled for by modelling it as a covariate effect, so that twin correlations would not be inflated due to pairs being the same age.

An assumption of the twin design is that trait-relevant environments are equally similar for MZ and DZ twins. Tests of this assumption suggest it is valid for personality [40], [41] and sexual orientation [42], so it seems a reasonable assumption for these mate preferences – indeed, it is not easy to see how mate-preference-relevant environmental factors would differ in similarity for MZ and DZ twins in ways that were not simply due to the greater genetic similarity of MZs (these kinds of differences would not violate the ‘equal-environments’ assumption (see [43]). Further details of the twin design, including assumptions, can be found elsewhere [38], [39].

## Results

Descriptives and age effects for the forced-choice mate preferences are shown in Table 1. The twin pair correlations in Table 2 show that twin pairs tended to hold somewhat similar preferences, indicating familial (i.e. genetic and/or shared environmental) influences. Averaged across traits, MZ twin pairs’ preferences correlated twice as strongly as DZ twin pairs in both men and women, corresponding to the difference in genetic relatedness of MZ and DZ pairs; this pattern of correlations strongly suggests genetic influences. In multivariate models, equating the twin correlations across traits significantly worsened the model fit for both males (p = .03) and females (p<.001), indicating significant variability between the variance components estimates for different trait preferences. Accordingly, genetic modelling results in Table 3 show that broad-sense heritability estimates (i.e. proportion of variation accounted for by all genetic factors; A+D) varied widely between the different trait preferences, ranging up to 48% for women’s preference for hair length and up to 57% for men’s preference for height. In the large female sample, all six trait preferences showed significant familial influences (i.e. E confidence intervals do not include 1.00), and four of the trait preferences showed significant genetic influences (i.e. A+D does not include 0.00). In the much smaller male sample, two of the five trait preferences (height and breast size) showed significant familial influences and only one (height) showed significant genetic influences. In either sex, only women’s preference for skin colour showed significant shared environmental influences.

**Table 2:** Twin pair correlations (and 95% confidence intervals) for dichotomous mate preferences for morphological traits.

	N (pairs)	Height	Skin colour	Hair colour	Hair length	Chest hair	Facial hair	Breast size	Overall(95%CI)
**MZF**	646–719	0.31 (0.13–0.48)	0.28 (0.09–0.46)	0.28 (0.14–0.41)	0.48 (0.38–0.58)	0.32 (0.20–0.43)	0.38 (0.21–0.53)	–	0.36 (0.30–0.41)
**DZF**	622–675	0.12 (−0.10–0.34)	0.41 (0.22–0.57)	−0.12 (−0.27–0.04)	0.18 (0.06–0.30)	0.29 (0.17–0.41)	0.11 (−0.06–0.27)	–	0.17 (0.11–0.23)
**MZM**	78–86	0.57 (0.27–0.79)	0.32 (−0.04–0.62)	−0.02 (−0.35–0.32)	0.18 (−0.16–0.50)	–	–	0.48 (0.16–0.73)	0.32 (0.16–0.46)
**DZM**	62–68	0.28 (−0.11–0.60)	−0.10 (−0.43–0.25)	−0.02 (−0.37–0.35)	0.23 (−0.21–0.60)	–	–	0.52 (0.17–0.78)	0.16 (−0.01–0.33)

‘Overall’ correlations are estimated by equating correlations across all traits in a multivariate model.

**Table 3:** Estimates (and 95% confidence intervals) of the proportion of variance in mate preferences accounted for by additive genetic (A), nonadditive genetic (D), family environmental (C), and residual (E) influences.

		Height	Skin colour	Hair colour	Hair length	Chest hair	Facial hair	Breast size
**Females**	A	0.18 (0.00–0.46)	0.00 (0.00–0.33)	0.00 (0.00–0.24)	0.24 (0.00–0.54)	0.06 (0.00–0.38)	0.06 (0.00–0.47)	–
	D	0.13 (0.00–0.48)	–	0.25 (0.00–0.38)	0.24 (0.00–0.57)	–	0.32 (0.00–0.53)	
	**A+D**	**0.31 (0.14–0.48)**	**0.00 (0.00–0.33)**	**0.25 (0.11–0.38)**	**0.48 (0.38–0.58)**	**0.06 (0.00–0.38)**	**0.38 (0.21–0.53)**	
	C	–	0.35 (0.07–0.47)	–	–	0.26 (0.00–0.37)	–	–
	E	0.69 (0.52–0.86)	0.65 (0.52–0.79)	0.75 (0.62–0.89)	0.52 (0.42–0.62)	0.68 (0.57–0.78)	0.62 (0.47–0.79)	–
**Males**	A	0.53 (0.00–0.78)	0.00 (0.00–0.49)	0.00 (0.00–0.29)	0.00 (0.00–0.50)	–	–	0.00 (0.00–0.70)
	D	0.04 (0.00–0.78)	0.28 (0.00–0.59)	–	–			–
	**A+D**	**0.57 (0.29–0.79)**	**0.28 (0.00–0.59)**	**0.00 (0.00–0.29)**	**0.00 (0.00–0.50)**			**0.00 (0.00–0.70)**
	C	–	–	0.00 (0.00–0.23)	0.20 (0.00–0.45)	–	–	0.50 (0.00–0.69)
	E	0.43 (0.21–0.71)	0.72 (0.41–1.00)	1.00 (0.71–1.00)	0.80 (0.50–1.00)	–	–	0.50 (0.27–0.73)

Height itself is highly heritable [44] and individuals’ preference for height in a mate depends to an extent on their own height [12]; as such, our estimate of heritability of height preference might simply reflect the heritability of height itself. To test this, we controlled for twins’ own height (available for a subsample of the total sample, N = 3524) by modeling it as a covariate in the variance components model, and re-estimated heritability. In this subsample, controlling for the twin’s own height reduced the heritability estimate for females from 23% to 14%, and for males from 66% to 52%, suggesting that height preference is heritable (at least in men) independent of its relationship with height itself.

We also tested whether variance components estimates of mate preferences differed between women of normal reproductive age (40 and under, when mate preferences are most consequential), and those over 40. For hair length preference, twin correlations (and hence variance components) differed significantly (p = .04) between women below the age of 40 (H^2^ = 56%) and those over 40 (H^2^ = 39%), but no other preference showed a significant difference between the two groups (all p>.1). The male sample was too small for a similar age comparison.

## Discussion

All of the trait preferences (height, skin colour, hair length and colour, chest and facial hair, and breast size) varied in the study population, and for all trait preferences this variation was due to significant familial effects in one or both sexes. In general, these familial effects were primarily genetic, with highly significant broad-sense heritability observed for a number of trait preferences. While our design did not afford sufficient power to statistically distinguish between additive and nonadditive genetic effects, it is generally implausible for complex traits to have nonadditive genetic variation in the complete absence of additive genetic variation [45], so our findings are consistent with the possibility raised by Darwin that ‘certain tastes’ for human beauty can be inherited. Regardless of the mode of inheritance, our finding of genetic influences bolsters sexual selection explanations for various morphological features because it provides a mechanism by which members of a population could tend to prefer certain morphological features over evolutionary timescales. It should be noted, though, that most of the variation was unexplained by genetic or family environmental influences, leaving much room for fickleness or idiosyncrasy in preferences.

It is worth noting the strongly sexually dimorphic preferences for height and skin colour (see Table 1). As to be expected, the vast majority of women prefer tall men to short men, whereas men’s preferences were evenly split; furthermore, height preference was significantly heritable in both men and women. These findings are consistent with a role for intersexual selection in sexual dimorphism in human height. Preference for skin colour was even more sexually dimorphic, but the vast majority of women preferred fair skin whereas most men preferred olive skin – this sex difference is in the opposite direction to that expected from sexual selection accounts in which evolution of lighter skin is supposed to be driven primarily by men preferring lighter skinned women [2], [3]. This unexpected finding could reflect a population-specific perceived association between skin colour and race and/or social class; interestingly, there was a significant family environmental influence on women’s skin colour preference but no significant genetic influence on either men or women’s preference. While not constituting strong evidence against the aforementioned sexual selection explanations of human skin colour variation, these findings seem to complicate matters somewhat. Moreover, recent research on skin colour and sexual attractiveness focuses on the role of carotenoids and the red/yellow continua [46], preferences for which would not have been picked up with our crude dichotomous measure.

Across the different trait preferences, heritability estimates ranged from 6% to 48% in women and from zero to 57% in men. This heterogeneity of variance component estimates was significant in both men and women, but the wide confidence intervals around the individual estimates should be kept in mind when interpreting the findings for particular traits, especially in men. The wide confidence intervals are in part because the measures are dichotomous (and thus imprecise), but are also exacerbated because when only twins raised together are available, since there is limited power to distinguish between family environmental and genetic influences. (Estimates of the total magnitude of familial influences on the traits are much more precise because these do not suffer from the partial confounding of genetic and family environmental influences.) Nevertheless, while there were no obvious patterns, it is worth checking for clues by considering which traits preferences had the highest heritabilities. For men, preference for height was easily the most heritable - this appears to only partly reflect the high heritability of height itself, since when individuals’ own height was used as a covariate the heritability of height preference was only a little lower. For women, the two preferences with the highest heritability were for hair length (long/short) and facial hair (beard or moustache/clean shaven), which are presumably the least heritable (within-population) of the target traits (Caucasians normally cannot directly inherit short hair or a clean shaven face). Because these grooming-related traits are environmentally malleable, preferences for them may be under weaker selection, which would be consistent with their high heritability (since strong selection tends to reduce heritable variation [7]). More mate preferences, more precise measures, and larger samples need to be subject to genetic analysis if we are to understand how the etiology of preferences relates to the etiology of the preferred traits. Genetic correlations between preferences and corresponding preferred traits are of particular interest because they are predicted by all mate choice models whenever there is genetic variation in both preference and preferred trait [47].

It should be noted that the demonstration of heritability of a preference does not suggest the existence of genes that code directly or specifically for that preference. Widespread pleiotropy (i.e. genes affecting multiple traits) is expected for genes underlying complex traits [48], [49], and the genetic influences on a given trait preference may overlap partially or fully with genetic influences on other traits such as broader mate preference dimensions, personality dimensions, or expression of the preferred trait itself.

Limitations inherent to the classical twin design warrant caution in interpreting our parameter estimates; in particular, separate estimates of additive and nonadditive genetic variance components are imprecise and subject to bias when using only twins, but estimates of the total genetic effect (i.e. broad-sense heritability) should be quite robust [50], [51]. Another limitation is the relatively small sample of men, which resulted in very imprecise variance components estimates, and the crude measurement of preferences, which would have introduced additional error variance (hence lowering the proportion of variation due to familial effects). Further, the findings are limited to one fairly homogenous population (British Caucasians) and do not explain the source of differences or similarities in preferences between populations. Lastly, the relatively old mean age of the sample raises questions about the extent to which these variance component estimates can be generalised across ages and cohorts, particularly given the seemingly fickle nature of trends in fashion and beauty. However, we do show that the estimates do not differ greatly between women under 40 and over 40 years of age. Overall, our findings show that mate preferences for specific morphological traits tend to run in families, mostly due to genetic factors, which provides an important reference point for sexual selection explanations of those morphological traits.
